# Real-world performance of point-of-care vs. standard-of-care HIV viral load testing in western Kenya: Secondary analysis of Opt4Kids and Opt4Mamas studies

**DOI:** 10.1371/journal.pgph.0003378

**Published:** 2024-06-24

**Authors:** Jessica H. Giang, Garoma Basha, Katherine K. Thomas, Patrick Oyaro, Bhavna H. Chohan, Leonard Kingwara, Shukri A. Hassan, Nashon Yongo, James Wagude, Fredrick Oluoch, Francesca Odhiambo, Boaz Oyaro, Grace C. John-Stewart, Lisa L. Abuogi, Rena C. Patel

**Affiliations:** 1 School of Medicine, University of Washington, Seattle, Washington, United States of America; 2 Department of Medicine, University of Washington, Seattle, Washington, United States of America; 3 Department of Global Health, University of Washington, Seattle, Washington, United States of America; 4 Health Innovations Kenya, Nairobi, Kenya; 5 National HIV Reference Laboratory, Kenya Ministry of Health, Nairobi, Kenya; 6 UWKenya, Nairobi, Kenya; 7 Department of Health, Siaya County, Kenya; 8 Department of Health, Kisumu County, Kenya; 9 Family AIDS Care and Education Services, Kenya Medical Research Institute, Kisumu, Kenya; 10 Kenya Medical Research Institute-CDC, Kisian, Kenya; 11 Departments of Pediatrics and Epidemiology, University of Washington, Seattle, Washington, United States of America; 12 Department of Pediatrics, University of Colorado, Denver, Colorado, United States of America; 13 Department of Medicine, University of Alabama at Birmingham, Birmingham, Alabama, United States of America; University of Georgia College of Public Health, UNITED STATES

## Abstract

Routine HIV viral load testing is important for evaluating HIV treatment outcomes, but conventional viral load testing has many barriers including expensive laboratory equipment and lengthy results return times to patients. A point-of-care viral load testing technology, such as GeneXpert HIV-1 quantification assay, could reduce these barriers by decreasing cost and turnaround time, however real-world performance is limited. We conducted a secondary analysis using 900 samples collected from participants in two studies to examine the performance of GeneXpert as point-of-care viral load compared to standard-of-care testing (which was conducted with two centralized laboratories using traditional HIV-1 RNA PCR quantification assays). The two studies, Opt4Kids (n = 704 participants) and Opt4Mamas (n = 820 participants), were conducted in western Kenya from 2019–2021 to evaluate the effectiveness of a combined intervention strategy, which included point-of-care viral load testing. Paired viral load results were compared using four different thresholds for virological non-suppression, namely ≥50, ≥200, ≥400, ≥1000 copies/ml. At a threshold of ≥1000 copies/mL, paired samples collected on the same day: demonstrated sensitivities of 90.0% (95% confidence interval [CI] 68.3, 98.8) and 66.7% (9.4, 99.2), specificities of 98.4% (95.5, 99.7) and 100% (96.5, 100), and percent agreements of 97.7% (94.6, 99.2) and 99.1% (95.0, 100) in Opt4Kids and Opt4Mamas studies, respectively. When lower viral load thresholds were used and the paired samples were collected an increasing number of days apart, sensitivity, specificity, and percent agreement generally decreased. While specificity and percent agreement were uniformly high, sensitivity was lower than expected. Non-specificity of the standard of care testing may have been responsible for the sensitivity values. Nonetheless, our results demonstrate that GeneXpert may be used reliably to monitor HIV treatment in low- and middle- income countries to attain UNAID’s 95-95-95 HIV goals.

## Introduction

Kenya has an estimated 1.3 million adults and 139,000 children living with HIV (CLWH). Among those on HIV antiretroviral treatment (ART), approximately 88.4% of adults but only 67.1% of children achieve viral suppression (VS) [1]. Routine viral load (VL) testing is needed to monitor HIV treatment and detect treatment failure, to prevent morbidity and mortality, and is recommended by the World Health Organization (WHO), including for low- and middle-income countries (LMICs) [2]. Kenya has long supported VL testing and has even increased frequency of VL monitoring among certain vulnerable subpopulations, such as CLWH and pregnant and postpartum women living with HIV [3, 4].

In Kenya, VL testing is conducted in centralized laboratories that require experienced laboratory personnel, specialized laboratory equipment, and transport of the specimens to the laboratories. This often leads to long turnaround and results return times to patients and infrequent VL testing due to the time and cost. Point-of-care (POC) VL testing has been shown to decrease both the cost of VL testing in medium and high volume clinics and turnaround times from 7–10 days to less than 1 day which can increase access and frequency of VL testing [5, 6].

One POC testing platform is the GeneXpert technology, which was originally developed for tuberculosis diagnostics in 2010 [6]. It has since been expanded for PCR amplification of RNA and DNA for many infectious pathogens, including HIV-1. The benefits of the GeneXpert platform for HIV-1 VL testing include a 90-minute runtime, an automated test process, and without a separate PCR room. GeneXpert HIV-1 VL testing has been validated by the company and others [7, 8], including in LMICs [6, 9–14].

While the GeneXpert testing for HIV-1 VL quantification has been validated for technical performance, there is limited data about its real-world performance. Our group conducted two related studies, Opt4Kids and Opt4Mamas, among CLWH and pregnant and postpartum women with HIV in Kenya using POC VL testing via the GeneXpert platform. Thus, we leveraged already collected data from these studies to analyze real-world performance of GeneXpert HIV VL testing compared to the standard of care (SOC) HIV VL testing. We hypothesized that GeneXpert HIV VL testing would demonstrate high sensitivity, specificity, and percent agreement in detecting lack of VS compared to the SOC HIV VL testing.

## Methods

### Study setting and population

We conducted a secondary, retrospective analysis of data collected in two related studies named Opt4Kids and Opt4Mamas conducted in Kisumu County, Kenya. Kisumu County has an adult HIV prevalence rate of 17.5% compared to Kenya’s national average of 4.9% [1]. Kisumu’s pediatric HIV prevalence is the second highest in the country with an estimated 9000 CLWH in Kisumu County [1, 15].

### Overview of parent studies Opt4Mamas and Opt4Kids

Opt4Kids study was an open label randomized control trial that explored the impact of POC VL testing, combined with targeted drug resistance mutation (DRM) testing and clinical decision support, on VS among CLWH on ART [16, 17]. The study enrolled 704 CLWH aged 1–14 in five high-volume treatment centers in Kisumu city and followed them for 12 months from March 2019 to December 2020. Participants were randomly assigned to intervention or SOC groups. The intervention group involved POC VL every 3 months using GeneXpert technology, targeted DRM testing when HIV VL ≥1000 copies/mL, and clinical support for interpretation of DRM testing results. The control group followed national guidelines with VL testing every 6 months and DRM testing after centralized review by the Regional NyaWest Technical Working Group.

Opt4Mamas study was a prospective cohort study that examined VS rates pre- and post- implementation of POC VL testing in pregnant and postpartum women living with HIV on ART [18]. The study recruited 820 pregnant women in the same five facilities as Opt4Kids during their antenatal care visits and followed them for six months postpartum from February 2019 to October 2021. For the pre-intervention group in the study, all the participants were given SOC throughout their care. After the intervention implementation, a new cohort of women was enrolled and underwent POC VL testing every 3 months, targeted DRM testing, and clinical decision support as described above for Opt4Kids.

### Sample collection and processing

Samples from Opt4Kids were collected from March 2019 to December 2020 and Opt4Mamas from February 2019 to August 2021. When routine care visits coincided with study visits, study staff attempted to coordinate blood collection from that participant for both routine care and study visit purposes from one venipuncture but with separate phlebotomy tubes. The samples were centrifuged on-site by the local laboratory technicians and who then aliquoted the plasma into vials appropriate for specimen testing. The testing plasma samples were kept at room temperature then sent for the respective SOC and POC VL testing within six hours of sample collection. However, this did not occur in all participants and it was not possible in all cases to discern if samples were collected via the same venipuncture for both SOC and POC VL testing if conducted on the same day. When the study visits could not be coordinated with the routine care visits or when the VL testing schedule differed between the study and routine care timelines, separate blood samples were obtained separately by study or routine care staff and sent for their respective testing. Although multiple samples were collected from the same individual, only one sample per participant was used in this study for analysis. The sample chosen was based on the shortest window of time between the POC and SOC VL results. Sample data were entered and stored in a REDCap project, alongside all study data, and data for this analysis were accessed in September 2022. The lead investigators for the Opt4Kids and Opt4Mamas studies had access to information that could identify individual participants after data collection though our analytic datasets contained de-identified information only.

### HIV-1 RNA quantification

SOC HIV-1 VL quantification was performed by Academic Model Providing Access To Healthcare (AMPATH) Care Laboratory or Kenya Medical Research Institute / Centers for Disease Control and Prevention (KEMRI/CDC) Kisian Laboratory on fresh plasma samples within 24–48 hours of blood collection. Occasionally the laboratories would freeze the plasma samples at -80°C in order to achieve sufficient batch volumes for testing, but this was not routinely documented. Both laboratories use a combination of automated real-time PCR nucleic acid amplification platforms, including Roche C6800 or C8800, Abbott M2000, and Hologic Panther, to conduct SOC HIV-1 VL testing, and staff were not aware of any POC VL test results. At these laboratories, the samples generally undergo an internal standard operating procedure for certain rejection criteria, e.g., hemolysis, clotting, sample identification mismatch, etc. Internal controls are included in each test run and periodic external quality control and assurance samples are also run.

We performed POC HIV-1 VL quantification via GeneXpert HIV-1 VL quantification assay via routine care laboratory staff, who were not aware of any of the SOC VL test results, at the study sites with study resources (e.g., study purchased cartridges and renumerated laboratory staff) on fresh plasma samples within the same day of sample collection. For the duration of the study, we participated in quarterly external quality control/assurance testing at all participating sites. When indeterminate or invalid results occurred, another vial of the same sample was used to run another POC VL test.

### Statistical analysis

We provided descriptive statistics of baseline characteristics of Opt4Kids and Opt4Mamas participant data. Our diagnostic outcome was lack of VS at varying VL cutoffs, ranging from HIV-1 VL ≥50, 200, 400, and 1000 copies/mL. The outcome was assessed concurrently on samples collected the same day (+/- 0 day) for both the POC and SOC VL testing. In secondary analyses, we broadened the time windows to include samples collected within 30 days of one another (i.e., +/- 30 days) and 90 days of one another (i.e., +/- 90 days), with each broader time window including the prior one.

We visually depicted the concordance of the two test results via scatter plots and reported diagnostic performance measures of sensitivity, specificity, and percent agreement (PA) with 95% confidence intervals (95% CI) for the POC VL test result using the SOC VL test result as the reference or “gold” standard. We generated confidence intervals using Clopper-Pearson method. We cleaned and analyzed the data using R version 4.2.3. We did not calculate any *a priori* sample sizes.

### Ethics review and informed consent

We obtained ethical approval for both studies from the African Medical and Research Foundation (AMREF) and Jaramogi Oginga Odinga Teaching and Referral Hospital (JOOTRH) Institutional Review Boards (IRBs) in Kenya, as well as the University of Washington and the University of Colorado Denver IRBs in the United States (with Opt4Kids registered as a trial, number NCT03820323). For Opt4Kids, study staff at each facility approached potential participants’ primary caregivers (usually a parent or guardian) for study participation at routine clinical visits and obtained written informed consent, with additional assent only for participants 13–14 years of age. For Opt4Mamas, study staff used similar approach but only obtained written informed consent from the participant.

## Results

### Participant characteristics

A total of 506 children and 394 women contributed data for this analysis (**Table 1**). For the children, at enrollment, the median age was 10 years (IQR 7, 12), their median time on ART was 6 years (3, 9), 54.0% were on non-nucleoside reverse transcriptase inhibitor (NNRTI)-containing ART, 67.2% of them at WHO clinical stage of I or II, and 73.5% of them had VS (defined as VL <1000 copies/mL). For the women, at enrollment, the median age was 30 years (26, 34), 50.0% had an education level of secondary school or above, median time on ART was 4 years (1, 7), 71.1% were on NNRTI-containing ART, 86.3% were WHO clinical stage I or II, and 77.4% virally suppressed. No adverse events related to VL testing occurred in either study.

**Table 1:** Baseline characteristics of Opt4Kids study, March 2019- December 2020, and Opt4Mamas study, February 2019 to August 2021, included in analysis.

Variable	Opt4Kids study (n = 506)	Opt4Mamas study (n = 394)
**Age at ART initiation in years (median, IQR)**	**2 (1, 5)**	**25 (22,29)**
**Age (median, IQR)**	**10 (7, 12)**	**30 (26, 34)**
**Time on ART in years (median, IQR)**	**6 (3, 9)**	**4 (1, 7)**
**Time on ART, n (%)**		
**<2 years**	**69 (13.6)**	**102 (26.0)**
**2–5 years**	**133 (26.3)**	**157 (39.8)**
**≥ 5 years**	**298 (59.0)**	**127 (32.2)**
**Unknown/missing**	**6 (1.2)**	**8 (2.0)**
**ART regimen, n (%)**		
**NNRTI-containing**	**273 (54.0)**	**280 (71.1)**
**PI-containing**	**211 (41.7)**	**33 (8.4)**
**Integrase-containing**	**22 (4.3)**	**56 (14.2)**
**Missing/Other**	**0**	**25 (6.3)**
**WHO clinical stage, most recent recorded within prior two years or on day of enrollment n (%)**		
**I or II**	**340 (67.2)**	**340 (86.3)**
**III or IV**	**121 (24.0)**	**37 (9.3)**
**Not indicated or missing**	**45 (8.9)**	**17 (4.3)**
**Food insecurity, n (%)**		
**No**	**101 (20.0)**	**147 (37.3)**
**Yes**	**405 (80.0)**	**247 (62.7)**
**Missing**	**0**	**1 (0.1%)**
**Education level, n (%)**		
**No education**	**1 (0.2)**	**7 (1.8)**
**Nursery/KG**	**82 (16.2)**	**0**
**Primary**	**379 (75.0)**	**190 (48.2)**
**Secondary & above**	**4 (0.8)**	**197 (50.0)**
**Missing**	**40 (8.0)**	**0**
**Viral suppression (<1000 copies/mL via SOC), as closest VL prior to two years to or on day of enrollment1, n (%)**		
**Yes**	**372 (73.5)**	**305 (77.4%)**
**No**	**112 (22.1)**	**40 (10.2%)**
**Missing**	**22 (4.3)**	**49 (12.4%)**
**Depression, n (%)**		
**Yes**	**79 (15.6)**	**273 (69.3)**
**No**	**426 (84.2)**	**120 (30.5)**
**Missing**	**1 (0.2)**	**1 (0.3)**

Abbreviations: SOC = standard-of-care; VL = Viral load; IQR = Inter quartile range; WHO = World Health Organization; ART = antiretroviral therapy; n = number of samples; KG = Kindergarten

1; NNRTI = non-nucleoside reverse transcriptase inhibitor; PI = protease inhibitor

### Sample characteristics

In total 900 samples were included from participants, 506 samples from Opt4Kids and 394 samples from Opt4Mamas. From the Opt4Kids study, there were 213 samples taken the same day, 345 samples taken within 30 days, and 506 samples taken within 90 days. From the Opt4Mamas, there were 106 samples taken the same day, 241 samples taken within 30 days, and 394 samples taken within 90 days.

### Evaluation of POC VL testing from Opt4Kids samples

**Fig 1:**
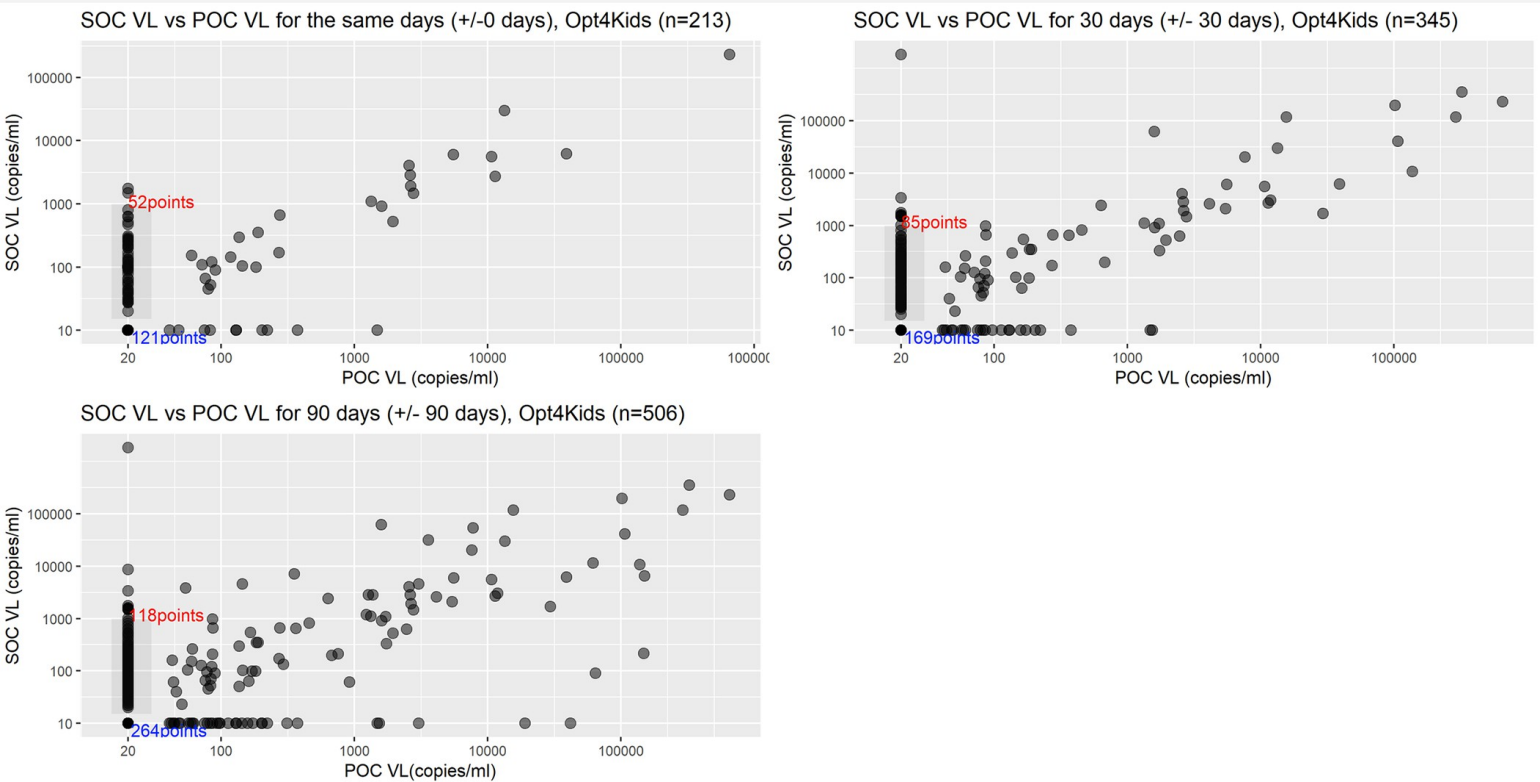
Scatter plots for POC vs. SOC VL test results by testing windows for Opt4Kids. Abbreviations: POC = point-of-care; SOC = standard-of-care; VL = viral load. The grey box in each plot delineates the range of VL values resulted by the SOC VL test when the POC VL was <40 copies/mL (lower limit of quantification). The red text indicates the number of such instances. The blue text indicates the number of instances where both the POC and SOC VL test results were lower than the limit of quantification.

Overall, concordance between the POC and SOC VL test results were qualitatively high **(Fig 1)** and specificity and percent agreement were high and improved with increased threshold for viral non-suppression, while sensitivity was modest (**Table 2**). For samples collected the same day (+/- 0 day) with VL≥1000 copies/mL, the sensitivity, specificity, and percent agreement were 90.0% (95% confidence interval 68.3, 98.8), 98.4% (95.5, 99.7), and 97.7% (94.6, 99.2), respectively. Sensitivity varied from 46.0% to 73.3%, specificity varied from 91.5% to 99.0%, and percent agreement varied from 74.2% to 95.3% for VL thresholds ≥50 to ≥400 copies/mL, generally increasing as threshold increased.

**Table 2:** Sensitivity, specificity, and percent agreement for Opt4Kids samples comparing POC vs. SOC VL testing using varying time differences and VL cutoff thresholds.

	SOC VL test positives		SOC VL test negatives		Percent Agreement (95% CI)
VL cutoff threshold (copies/mL)	n/N (%) positive by POC (TP)	Sensitivity (95% CI)	n/N (%) negative by POC (TN)	Specificity (95% CI)	
***Same day (+/- 0 day)*, *n = 213 samples***					
VL≥50	39/83	47.0% (36.0, 58. 3)	119/130	91.5% (85.4, 95.7)	74.2% (67.8, 80.0)
VL≥200	23/50	46.0% (31.8, 60.7)	157/163	96.3% (92.2, 98.6)	84.5% (79.0, 89.1)
VL≥400	22/30	73.3% (54.1, 87.7)	181/183	99.0% (96.1, 99.9)	95.3% (91.5, 97.7)
VL≥1000	18/20	90.0% (68.3, 98.8)	190/193	98.4% (95.5, 99.7)	97.7% (94.6, 99.2)
***30 days (+/- 30 days)*, *n = 345 samples***					
VL≥50	54/128	42.2% (33.5, 51.2)	197/217	90.8% (86.1, 94.3)	72.8% (67.7, 77.4)
VL≥200	32/74	43.2% (31.8, 55.3)	264/271	97.4% (94.8, 99.0)	85.8% (81.7, 89.3)
VL≥400	29/50	58.0% (43.2, 71.8)	291/295	98.6% (96.6, 99.6)	92.8% (89.5, 95.3)
VL≥1000	24/33	72.7% (54.5, 86.7)	306/312	98.1% (95.9, 99.3)	95.7% (93.0, 97.5)
***90 days (+/- 90 days)*, *n = 506 samples***					
VL≥50	72/177	40.7% (33.4, 48.3)	300/329	91.2% (87.6, 94.0)	73.5% (69.4, 77.3)
VL≥200	43/96	44.8% (34.6, 55.3)	395/410	96.3% (94.0, 98.0)	86.6% (83.3, 89.4)
VL≥400	37/64	57.8% (44.8, 70.1)	431/442	97.5% (95.6, 98.8)	92.5% (89.8, 94.6)
VL≥1000	32/44	72.7% (57.2, 85.0)	451/462	97.6% (95.8, 98.8)	95.5% (93.3, 97.1)

Abbreviations: POC = point-of-care; SOC = standard-of-care; VL = Viral load; TP = true positive; TN = true negative

Specificity and percent agreement remained relatively high when comparing samples collected within +/- 30 and 90 days, while sensitivity remained modest (**Table 2**). For samples collected +/- 30 days, the sensitivity, specificity, and percent agreement were 72.7% (54.5, 86.7), 98.1% (95.9, 99.3), and 95.7% (93.0, 97.5) for VL≥1000 copies/mL, respectively. Sensitivity varied from 42.2% to 58.0%, specificity varied from 90.8% to 98.6%, and percent agreement varied from 72.8% to 92.8% for VL thresholds ≥50 to ≥400 copies/mL, generally increasing as threshold increased. For samples collected +/-90 days, the sensitivity, specificity, and percent agreement were 72.7% (57.2, 85.0), 97.6% (95.8, 98.8), and 95.5% (93.3, 97.1) for VL≥1000 copies/mL, respectively. Sensitivity varied from 40.7% to 57.8%, specificity varied from 91.2% to 97.5%, and percent agreement varied from 73.5% to 92.5% for VL thresholds ≥50 to ≥400 copies/mL, also increasing as threshold increased.

### Evaluation of POC VL testing from Opt4Mamas samples

**Fig 2:**
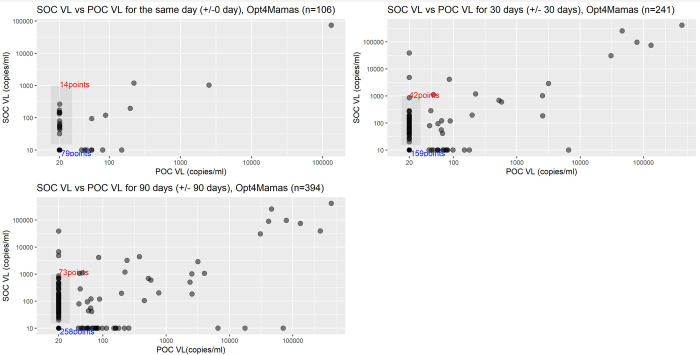
Scatter plots of POC vs. SOC VL test results by testing windows for Opt4Mamas. Abbreviations: POC = point-of-care; SOC = standard-of-care; VL = viral load. The grey box in each plot delineates the range of VL values resulted by the SOC VL test when the POC VL was <40 copies/mL (lower limit of quantification). The red text indicates the number of such instances. The blue text indicates the number of instances where both the POC and SOC VL test results were lower than the limit of quantification.

Similarly, to the Opt4Kids samples, overall concordance between the POC and SOC VL test results were qualitatively high **(Fig 2)**, and specificity and percent agreement improved with increasing the viral load threshold, while sensitivity was modest for Opt4Mamas samples (**Table 3**). For samples collected the same day, the sensitivity, specificity, and percent agreement were 66.7% (9.4, 99.2), 100% (96.5, 100), and 99.1% (95.0, 100) for VL≥1000 copies/mL. Sensitivity varied from 35.3% to 75.0%, specificity varied from 95.5% to 100%, and percent agreement varied from 85.8% to 99.1% for VL thresholds ≥50 to ≥400 copies/mL, generally increasing as threshold increased.

**Table 3:** Sensitivity, specificity, and percent agreement for Opt4Mamas samples comparing POC vs. SOC VL testing using varying time differences and VL cutoff thresholds.

	SOC VL test positives		SOC VL test negatives		Percent Agreement (95% CI)
VL cutoff threshold (copies/mL)	n/N (%) positive by POC (TP)	Sensitivity (95% CI)	n/N (%) negative by POC (TN)	Specificity (95% CI)	
***Same day (+/- 0 day)*, *n = 106 samples***					
VL≥50	6/17	35.3% (14.2, 61.7)	85/89	95.5% (88.9, 98.8)	85.8% (77.7, 91.9)
VL≥200	3/4	75.0% (19.4, 99.4)	102/102	100% (96.4, 100)	99.1% (95.0, 100.0)
VL≥400	2/3	66.7% (9.4, 99.2)	103/103	100% (96.6, 100)	99.1% (95.0, 100)
VL≥1000	2/3	66.7% (9.4, 99.2)	103/103	100% (96.5, 100)	99.1% (95.0, 100)
***30 days (i*.*e*., *+/- 30 days)*, *n = 241 samples***					
VL≥50	17/54	31.5% (19.5, 45.6)	174/187	93.0% (88.4, 96.2)	79.3% (73.6, 84.2)
VL≥200	10/20	50.0% (27.2, 72.8)	219/221	99.1% (96.8, 99.9)	95.0% (91.5, 97.4)
VL≥400	9/15	60.0% (32.3, 83.7)	224/226	99.1% (96.8, 99.9)	96.7% (93.6, 98.6)
VL≥1000	7/12	58.3% (27.7, 84.8)	227/229	99.1% (96.9, 99.9)	97.1% (94.1, 98.8)
***90 days (i*.*e*., *+/- 90 days)*, *n = 394 samples***					
VL≥50	25/90	27.8% (19.0, 38.2)	280/304	92.1% (88.5, 95.0)	77.4% (73.0, 81.4)
VL≥200	17/39	43.6% (27.8, 60.4)	348/355	98.0% (96.0, 99.2)	92.6% (89.6, 95.0)
VL≥400	13/30	43.3% (25.5, 62.6)	358/364	98.4% (96.4, 99.4)	94.2% (91.4, 96.3)
VL≥1000	10/19	52.6% (28.9, 75.6)	370/375	98.7% (97.0, 99.6)	96.4% (94.1, 98.0)

Abbreviations: POC = point-of-care; SOC = standard-of-care; VL = Viral load; TP = true positive; TN = true negative

Following the pattern above for Opt4Mamas samples, specificity and percent agreement remained relatively high when comparing samples collected within +/- 30 and 90 days, while sensitivity remained modest (**Table 3**). For samples collected +/- 30 days, the sensitivity, specificity, and percent agreement were 58.3% (27.7, 84.8), 99.1% (96.9, 99.9), and 97.1% (94.1, 98.8) for VL≥1000 copies/mL. Sensitivity varied from 31.5% to 60.0%, specificity varied from 93.0% to 99.1%, and percent agreement varied from 79.3% to 96.7% for VL thresholds ≥50 to ≥400 copies/mL, generally increasing as threshold increased. For samples collected +/-90 days, the sensitivity, specificity, and percent agreement were 52.6% (28.9, 75.6), 98.7% (97.0, 99.6), and 96.4% (94.1, 98.0) for VL≥1000 copies/mL. Sensitivity varied from 27.8% to 43.6%, specificity varied from 92.1% to 98.4%, and percent agreement varied from 77.4% to 94.2% for VL thresholds ≥50 to ≥400 copies/mL, generally increasing as threshold increased.

## Discussion

Our secondary analysis of samples collected for both POC and SOC VL testing among children and pregnant/postpartum women with HIV on treatment in Kenya suggests that the GeneXpert HIV-1 VL quantification platform generally performed well against the “gold standard” during this real-world implementation of POC VL testing. As expected, performance improved as the VL threshold cutoff for viral non-suppression increased. Nonetheless, even with low VL cutoffs, specificity (>90%) was high while percent agreement was moderately high (>70%) between POC and SOC VL testing. Performance was reduced as the number of days between the collection time of the two samples increased. Sensitivity was in the 90% range for the highest VL cutoff, but decreased to as low as the 30% range for the lowest or strictest threshold of VL cutoff.

Validation studies comparing the same POC platform of GeneXpert as we used but done in other countries had specificity and sensitivity generally being greater than 90% and percent agreement greater than 80% (see **S1 Table**). Colleagues in Botswana used fresh blood samples that were tested within 4 hours of collection with a VL thresholds of >40–1000 copies/mL reported sensitivities of 98.6 to 99.6% and percent agreement of 91–97% [12]. Similarly colleagues in India used frozen blood samples that were tested within 2 days of collection with VL thresholds of ≥40–500,000 copies/mL, their sensitivities were 97%, specificities 97–100%, and percent agreement 82–100% [13]. In a study done in Malawi, where colleagues used a VL threshold of ≥1000 copies/mL and samples collected within 90 days of each other, the calculated sensitivity, specificity, and percent agreement 92.2%, 91.7%, and 90.9%, respectively [8]. A meta-analysis comparing GeneXpert to SOC testing had a pooled sensitivity of 96.5% and specificity of 96.6% at a viral load threshold of 1000 copies/ml [19]. Despite minor differences in study design, VL thresholds, blood samples, or other elements, all the studies corroborate high concordance of GeneXpert with the gold standard.

Compared to our specificity and percent agreement values, our sensitivity values were lower than expected when compared to sensitivity values reported in other published validation studies of GeneXpert for POC HIV VL testing [6, 8, 12–14, 19–21]. Also interestingly, as VL cutoff values decreased from <1000 to <50 copies/mL, noting a trend in national and international guidelines to reduce the cutoff for determining viral suppression [2, 3], the sensitivity tended to decrease though specificity remained high. This is a likely acceptable tradeoff as identifying individuals not at risk for viremia more accurately could be considered better than missing individuals who are viremic; of course, additional costs of repeat VLs, counseling sessions, and other healthcare interventions for someone truly suppressed are also not trivial. As can be appreciated in our figures (Figs 1 and 2), many of the discordant values are when the POC VL test result is reported as undetectable or lower than limit of detection while the SOC VL test result has a quantifiable value, some of which fall into clinically and meaningfully different levels. This is interpreted as modest sensitivity values for the POC VL test, a few potential reasons may explain this finding. That the sensitivity decreased largely at low levels of viremia raises the concern that the low sensitivity could be due to cross contamination between samples due to high throughput testing in SOC VL testing settings. In other words, if some samples had undetectable VL but due to contamination with other samples with viremia, they erroneously become falsely positive, which would incorrectly render the POC result of undetectable as falsely negative. In a study done in South Africa and Zambia, the authors reported high specificity values but also lower than expected sensitivity values, and found that there were quality control issues in carrying out VL testing due to sample cross contamination [22]. Alternatively, it could also be possible that the SOC assays have higher sensitivity to detect VL at low levels, while the POC assay was indeed less sensitive at lower levels of viremia. Given greater variability in user training, test kit handling and storage, testing environments, and protocol of POC assays, these factors too could lower the sensitivity of the POC assay. Despite these possibilities, we do not have reason to believe the POC VL assay we used has some intrinsic limitation with VL detection at low levels of viremia, as this specific range has been interrogated in dedicated validation studies which show high sensitivity values [12, 13, 19, 21].

Nonetheless, given its overall reliability, GeneXpert HIV-1 VL testing may provide a useful measure of high threshold viral failure (≥1000 copies/mL) while decreasing cost and turnaround times [16]. The determination of usability of POC tests in the field goes beyond just sensitivity and specificity values, the WHO utilizes the REASSURED framework to evaluate POC tests. The framework criteria includes ease of specimen collection, affordability, user-friendliness, rapid results to return, and deliverability [23]. Drawing from our own qualitative work and other studies, as GeneXpert HIV-1 VL testing meets these criteria, we remain optimistic for the role of POC VL testing in LMIC [24–26]. The WHO recommends making POC VL tests as part of routine clinical care and prioritizing POC VL testing in high risk groups like children and pregnant/postpartum women living with HIV due to the need for prompt results [27]. Patients can receive VL testing results within the same day rather than wait for several days, which has significant value to both patients and providers [26]. With real-world implementation data of POC VL testing such as ours, patients, providers, and policy makers can have confidence in incorporating POC VL testing into standard HIV treatment guidelines in clinics across Kenya and other LMICs, especially at the higher VL thresholds. At lower VL thresholds, while the specificity remained high, the sensitivity dropped below 50% at times; arguably less harm is done to patients for the possibility of erroneously considering them viremic when they truly are not, though, as noted above, consequential costs do exist for these false positives. Anticipated challenges to widescale POC VL testing include obtaining and maintaining POC VL testing platforms for multiple clinics, training staff to use these platforms, optimizing testing platform network utilization via creating testing hubs, and ensuring quality control throughout multiple sites. Overall, with relatively high specificity and percent agreement, the comparison between the SOC VL testing and POC VL GeneXpert testing shows encouraging results and provides credence for integration of POC VL testing in routine care practices based on our real-world usage of POC VL testing in an LMIC setting.

Though our study utilizes real-world data with highest numbers of samples reported to date, there is one major limitation to this study. Our analysis was not conducted as an explicit validation study but rather as a secondary analysis that leveraged existing data from other studies; thus, there is potential for biases though we have not readily identified any. Ideally, for even a validation study using real-world implementation, there would have been one blood sample collected from the participant with one venipuncture and the specimen being split into two tubes for the two testing methods, and all samples would be from the same day. Nonetheless, our findings with real-world implementation provides additional evidence that the overall performance of POC VL testing is stable even across months, and mostly comparable to SOC. Additionally, that we detected low sensitivity, raising the concerns about potential compromises in the “gold standard” testing, is admittedly unsettling. Future studies, intentionally planned for validation purposes, can institute robust protocols under study purview to reduce such potential limitations.

## Conclusion

Real-world implementation of POC VL testing using the GeneXpert HIV-1 VL quantification testing platform, compared to SOC VL testing platforms in Kisumu, Kenya, showed high values for specificity and percent agreement and moderate for sensitivity at a VL threshold of 1000 copies/mL. At lower VL thresholds and wider time windows between sample collection, specificity and percent agreement remained relatively high, but sensitivity decreased considerably. This tradeoff may be acceptable while both POC and SOC VL testing techniques are optimized. The performance of POC testing supports its integration into routine VL monitoring and may contribute to efforts in Kenya and other LMIC to reach the UNAIDS 95-95-95 targets.

## Supporting information

S1 Table Summary of published validation studies comparing GeneXpert HIV-1 quantification assay vs. standard of care technology in low- and middle-income countries. (DOCX)

S2 Table Cross tabulation of POC vs. SOC VL test results, Opt4Kids (n = 704 total and n = 820 participants). (DOCX)

S1 File Paired viral load data for Opt4Kids and Opt4Mamas. (PDF)

## References

[1] National AIDS and STI Control Programme (NASCOP). *Preliminary KENPHIA 2018 Report Kenya Ministry of Health*, *Nairobi*, *Kenya*.; 2020.

[2] World Health Organization. Consolidated guidelines on the use of antiretroviral drugs for treating and preventing HIV infection: recommendations for a public health approach, 2nd ed. World Health Organization.

[3] NASCOP. *Ministry of Health*, *National AIDS & STI Control Program*. *Guidelines on Use of Antiretroviral Drugs for Treating and Preventing HIV Infection in Kenya 2018 Edition*, *Print*.; 2018.

[4] National AIDS & STI Control Programme (NASCOP). Kenya HIV Prevention and Treatment Guidelines.

[5] Ganesh P, Heller T, Chione B, Gumulira J, Gugsa S, Khan S, et al. Near Point-of-Care HIV Viral Load: Targeted Testing at Large Facilities. J Acquir Immune Defic Syndr. 2021 Feb 1;86(2):258–63. doi: 10.1097/QAI.0000000000002555 33136821 PMC7803448

[6] Nash M, Ramapuram J, Kaiya R, Huddart S, Pai M, Baliga S. Use of the GeneXpert tuberculosis system for HIV viral load testing in India. The Lancet Global Health. 2017 Aug;5(8):e754–5. doi: 10.1016/S2214-109X(17)30247-4 28716346

[7] Jordan JA, Plantier JC, Templeton K, Wu AHB. Multi-site clinical evaluation of the Xpert® HIV-1 viral load assay. Journal of Clinical Virology. 2016 Jul 1;80:27–32. 27135387 10.1016/j.jcv.2016.04.014

[8] Manavi K, Hodson J, Masuka S, Singo M, Dedicoat K, Osman H. Correlation between Cepheid GeneXpert and Abbott M2000 assays for HIV viral load measurements. Int J STD AIDS. 2021 Apr 1;32(5):444–8. doi: 10.1177/0956462420975606 33427080

[9] Ceffa S, Luhanga R, Andreotti M, Brambilla D, Erba F, Jere H, et al. Comparison of the Cepheid GeneXpert and Abbott M2000 HIV-1 real time molecular assays for monitoring HIV-1 viral load and detecting HIV-1 infection. Journal of Virological Methods. 2016 Mar;229:35–9. doi: 10.1016/j.jviromet.2015.12.007 26709099

[10] Ndoye AS, Ndiaye HD, Diallo M, Diack A, Coulibaly K, Lo G, et al. Validation of the point-of-care (POC) technologies Xpert HIV-1 Qual and m-PIMA HIV 1/2 detect for early diagnosis of HIV-1 and HIV-2 in Senegal. Pan Afr Med J. 2022;43:42. doi: 10.11604/pamj.2022.43.42.32714 36523274 PMC9733457

[11] Garrett NJ, Drain P, Werner L, Samsunder N, Karim SSA. Diagnostic Accuracy of the Point-of-care Xpert HIV-1 Viral Load Assay in a South African HIV clinic. J Acquir Immune Defic Syndr. 2016 Jun 1;72(2):e45–8. doi: 10.1097/QAI.0000000000000978 26959192 PMC4866899

[12] Moyo S, Mohammed T, Wirth KE, Prague M, Bennett K, Holme MP, et al. Point-of-Care Cepheid Xpert HIV-1 Viral Load Test in Rural African Communities Is Feasible and Reliable. J Clin Microbiol. 2016 Dec;54(12):3050–5. doi: 10.1128/JCM.01594-16 27733636 PMC5121399

[13] Kulkarni S, Jadhav S, Khopkar P, Sane S, Londhe R, Chimanpure V, et al. GeneXpert HIV-1 quant assay, a new tool for scale up of viral load monitoring in the success of ART programme in India. BMC Infect Dis. 2017 Jul 21;17:506. doi: 10.1186/s12879-017-2604-5 28732472 PMC5521114

[14] Bwana P, Ageng’o J, Mwau M. Performance and usability of Cepheid GeneXpert HIV-1 qualitative and quantitative assay in Kenya. PLoS One. 2019 Mar 22;14(3):e0213865. doi: 10.1371/journal.pone.0213865 30901343 PMC6430374

[15] Ministry of Health (MOH). Kenya HIV Estimates Report; 2020.

[16] Patel RC, Oyaro P, Thomas KK, Wagude J, Mukui I, Brown E, et al. Point-of-care HIV viral load and targeted drug resistance mutation testing versus standard care for Kenyan children on antiretroviral therapy (Opt4Kids): an open-label, randomised controlled trial. Lancet Child Adolesc Health. 2022 Oct;6(10):681–91. doi: 10.1016/S2352-4642(22)00191-2 35987208 PMC9482947

[17] Patel RC, Oyaro P, Odeny B, Mukui I, Thomas KK, Sharma M, et al. Optimizing viral load suppression in Kenyan children on antiretroviral therapy (Opt4Kids). Contemp Clin Trials Commun. 2020 Dec;20:100673. doi: 10.1016/j.conctc.2020.100673 33195874 PMC7644580

[18] Patel RC, Oyaro P, Thomas KK, Basha GW, Wagude J, Mukui I, et al. Impact of point‐of‐care HIV viral load and targeted drug resistance mutation testing on viral suppression among Kenyan pregnant and postpartum women: results from a prospective cohort study (Opt4Mamas). J Int AIDS Soc. 2023 Nov 8;26(11):e26182. doi: 10.1002/jia2.26182 37938856 PMC10631517

[19] Sacks JA, Fong Y, Gonzalez MP, Andreotti M, Baliga S, Garrett N, et al. Performance of Cepheid Xpert HIV-1 viral load plasma assay to accurately detect treatment failure. AIDS. 2019 Oct 1;33(12):1881–9. doi: 10.1097/QAD.0000000000002303 31274537 PMC7024604

[20] GeneXpert HIV-1 VL assay package insert. https://www.pei.de/SharedDocs/Downloads/DE/arzneimittelsicherheit/ivd-vigilanz/ivd-herstellermassnahmen/2018/PEI0091-18-en.pdf?__blob=publicationFile&v=2

[21] Ochodo EA, Olwanda EE, Deeks JJ, Mallett S. Point-of-care viral load tests to detect high HIV viral load in people living with HIV/AIDS attending health facilities. Cochrane Database Syst Rev. 2022 Mar 10;3(3):CD013208. doi: 10.1002/14651858.CD013208.pub2 35266555 PMC8908762

[22] Bock P, Phiri C, Piwowar-Manning E, Kosloff B, Mandla N, Young A, et al. Understanding low sensitivity of community-based HIV rapid testing: experiences from the HPTN 071 (PopART) trial in Zambia and South Africa. J Int AIDS Soc. 2017 Aug 29;20(Suppl 6):21780. doi: 10.7448/IAS.20.7.21780 28872272 PMC5625636

[23] Unemo M, Cole M, Lewis D, Ndowa F, Van Der Pol B, Wi T, et al. Laboratory and point-of-care diagnostic testing for sexually transmitted infections, including HIV. Geneva: World Health Organization; 2023.

[24] Qian SRW, Hassan SA, Scallon AJ, Oyaro P, Brown E, Wagude J, et al. “After viral load testing, I get my results so I get to know which path my life is taking me”: qualitative insights on routine centralized and point-of-care viral load testing in western Kenya from the Opt4Kids and Opt4Mamas studies. BMC Health Serv Res. 2022 Dec 17;22(1):1540. doi: 10.1186/s12913-022-08593-z 36528677 PMC9758673

[25] Sandbulte MR, Gautney BJ, Maloba M, Wexler C, Brown M, Mabachi N, et al. Infant HIV testing at birth using point-of-care and conventional HIV DNA PCR: an implementation feasibility pilot study in Kenya. Pilot Feasibility Stud. 2019;5:18. doi: 10.1186/s40814-019-0402-0 30701079 PMC6347792

[26] Rutstein SE, Golin CE, Wheeler SB, Kamwendo D, Hosseinipour MC, Weinberger M, et al. On the front line of HIV virological monitoring: barriers and facilitators from a provider perspective in resource-limited settings. AIDS Care. 2016;28(1):1–10. doi: 10.1080/09540121.2015.1058896 26278724 PMC4834050

[27] Updated recommendations on HIV prevention, infant diagnosis, antiretroviral initiation and monitoring: March 2021. Geneva: World Health Organization; 2021. 33822559

