# Supplementary material for: Real-world performance of point-of-care vs. standard-of-care HIV viral load testing in western Kenya: Secondary analysis of Opt4Kids and Opt4Mamas studies
Source: PLOS Glob Public Health. 2024 Jun 24;4(6):e0003378. doi: 10.1371/journal.pgph.0003378 (PMC11195974; doi:10.1371/journal.pgph.0003378)
Supplement: S1 Table — (DOCX) [file pgph.0003378.s001.docx]

**S1 Table: Summary of published validation studies comparing GeneXpert HIV-1 quantification assay vs. standard of care technology in low- and middle-income countries**

| **Study Authors** | **Country and Publication Year** | **Sample Size** | **Sample Characteristics** | **VL Threshold (copies/mL)** | **Sensitivity (%)** | **Specificity (%)** | **Percent Agreement (%)** | **SOC Technology** |
| --- | --- | --- | --- | --- | --- | --- | --- | --- |
| Ceffa et al. | Malawi, 2016 | 274 | Within 90 days | 1000 | Not reported (Calculated 92.2) | Not reported (Calculated 91.7) | 90.9 | Abbott Real-Time HIV-1 VL |
| Garrett et al. | South Africa, 2016 | 42 | Fresh and frozen samples | 1000 | Not reported | Not reported | Not reported (Calculated 97.6) | Roche TaqMan version 2 assay |
| Moyo et al. | Botswana, 2016 | 277 | Fresh blood tested within 4 hours of collection | 40-1000 | 98.6-99.6 | Not reported | 90.6-97.1 | Abbott m2000sp/m2000rt assay |
| Kulkarni et al. | India, 2017 | 219 | Frozen blood tested within 2 days of collection | 40 - >500,000 | 97 | 97-100 | 82-100 | Abbott m2000 Real Time PCR |
| Gous et al. | South Africa, 2016 | 158 | Fresh and frozen plasma | 1000 | 92.9-100 | 95.9-96.9 | Not reported | Roche Cobas TaqMan v2 and Abbott HIV-1 RT |
| Bwana et al. | Kenya, 2019 | 100 | Plasma samples | 1000 | 92.5 | 100 | Not reported | Abbott m2000 assay |
