# Supplementary material for: Real-world performance of point-of-care vs. standard-of-care HIV viral load testing in western Kenya: Secondary analysis of Opt4Kids and Opt4Mamas studies
Source: PLOS Glob Public Health. 2024 Jun 24;4(6):e0003378. doi: 10.1371/journal.pgph.0003378 (PMC11195974; doi:10.1371/journal.pgph.0003378)
Supplement: S2 Table — (DOCX) [file pgph.0003378.s002.docx]

**S2 Table: Cross tabulation of POC vs. SOC VL test results, Opt4Kids (n=704 total and n=820 participants)**

| **Same day (i.e., +/- 0 day, kids), n=213 samples** | | | **Total,**  **Percent agreement (95% CI)** |
| --- | --- | --- | --- |
| **VL>50** | | | |
|  | **SOC VL** | |  |
| **POC VL** | >50  Sensitivity (95% CI) | <50  Specificity (95% CI) |  |
| >50 | 39 | 11 | 50 |
| <50 | 44 | 119 | 163 |
| Total | 83  47.0% (36.0, 58. 3) | 130  91.5% (85.4, 95.7) | 213  74.2% (67.8, 80.0) |
|  | **SOC VL** | |  |
| **POC VL** | >200  Sensitivity (95% CI) | <200  Specificity (95% CI) |  |
| >200 | 23 | 6 | 29 |
| <200 | 27 | 157 | 184 |
| Total | 50  46.0% (31.8, 60.7) | 163  96.3% (92.2, 98.6) | 213  84.5% (79.0, 89.1) |
|  | **SOC VL** | | |
| **POC VL** | >400  Sensitivity (95% CI) | <400  Specificity (95% CI) |  |
| >400 | 22 | 2 | 24 |
| <400 | 8 | 181 | 189 |
| Total | 30  73.3% (54.1, 87.7) | 183  99.0% (96.1, 99.9) | 213  95.3% (91.5, 97.7) |
|  | **SOC VL** | |  |
| **POC VL** | >1000  Sensitivity (95% CI) | <1000  Specificity (95% CI) |  |
| >1000 | 18 | 3 | 21 |
| <1000 | 2 | 190 | 192 |
| Total | 20  90.0% (68.3, 98.8) | 193  98.4% (95.5, 99.7) | 213  97.7% (94.6, 99.2) |
| **Same day (i.e., +/- 0 day, mamas), n= 106 unique individuals** | | | |
| **VL>50** | | | |
|  | **SOC VL** |  |  |
| **POC VL** | >50  Sensitivity (95% CI) | <50  Specificity (95% CI) |  |
| >50 | 6 | 4 | 10 |
| <50 | 11 | 85 | 96 |
| Total | 17  35.3% (14.2, 61.7) | 89  95.5% (88.9, 98.8) | 106  85.8% (77.7, 91.9) |
| **VL>200** | | | |
|  | **SOC VL** | |  |
| **POC VL** | >200  Sensitivity (95% CI) | <200  Specificity (95% CI) |  |
| >200 | 3 | 0 | 3 |
| <200 | 1 | 102 | 103 |
| Total | 4  75.0% (19.4, 99.4) | 102  100% (96.4, 100) | 106  99.1% (95.0, 100.0) |
| **VL>400** | | | |
|  | **SOC VL** | |  |
| **POC VL** | >400  Sensitivity (95% CI) | <400  Specificity (95% CI) |  |
| >400 | 2 | 0 | 2 |
| <400 | 1 | 103 | 104 |
| Total | 3  66.7% (9.4, 99.2) | 103  100% (96.6, 100) | 106  99.1% (95.0, 100) |
|  | **SOC VL** | |  |
| **POC VL** | >1000  Sensitivity (95% CI) | <1000  Specificity (95% CI) |  |
| >1000 | 2 | 0 | 2 |
| <1000 | 1 | 103 | 104 |
| Total | 3  66.7% (9.4, 99.2) | 103  100% (96.5, 100) | 106  99.1% (95.0, 100) |
| **30 days (i.e., +/- 30 days, kids), n= 345 samples** | | | |
| **VL>50** | | | |
|  | **SOC VL** | |  |
| **POC VL** | >50  Sensitivity (95% CI) | <50  Specificity (95% CI) |  |
| >50 | 54 | 20 | 74 |
| <50 | 74 | 197 | 271 |
| Total | 128  42.2% (33.5, 51.2) | 217  90.8% (86.1, 94.3) | 345  72.8% (67.7, 77.4) |
| **VL>200** | | | |
|  | **SOC VL** | |  |
| **POC VL** | >200  Sensitivity (95% CI) | <200  Specificity (95% CI) |  |
| >200 | 32 | 7 | 39 |
| <200 | 42 | 264 | 306 |
| Total | 74  43.2% (31.8, 55.3) | 271  97.4% (94.8, 99.0) | 345  85.8% (81.7, 89.3) |
| **VL>400** | | | |
|  | **SOC VL** |  |  |
| **POC VL** | >400  Sensitivity (95% CI) | <400  Specificity (95% CI) |  |
| >400 | 29 | 4 | 33 |
| <400 | 21 | 291 | 312 |
| Total | 50  58.0% (43.2, 71.8) | 295  98.6% (96.6, 99.6) | 345  92.8% (89.5, 95.3) |
| **VL>1000** | | | |
|  | **SOC VL** | |  |
| **POC VL** | >1000  Sensitivity (95% CI) | <1000  Specificity (95% CI) |  |
| >1000 | 24 | 6 | 30 |
| <1000 | 9 | 306 | 315 |
| Total, | 33  72.7% (54.5, 86.7) | 312  98.1% (95.9, 99.3) | 345  95.7% (93.0, 97.5) |
| **30 days (i.e., +/- 30 days, mamas), n= 241 unique samples** | | | |
| **VL>50** | | | |
|  | **SOC VL** |  |  |
| **POC VL** | >50  Sensitivity (95% CI) | <50  Specificity (95% CI) |  |
| >50 | 17 | 13 | 30 |
| <50 | 37 | 174 | 211 |
| Total | 54  31.5% (19.5, 45.6) | 187  93.0% (88.4, 96.2) | 241  79.3% (73.6, 84.2) |
| **VL>200** | | | |
|  | **SOC VL** | |  |
| **POC VL** | >200  Sensitivity (95% CI) | <200  Specificity (95% CI) |  |
| >200 | 10 | 2 | 12 |
| <200 | 10 | 219 | 229 |
| Total | 20  50.0% (27.2, 72.8) | 221  99.1% (96.8, 99.9) | 241  95.0% (91.5, 97.4) |
| **VL>400** | | | |
|  | **SOC VL** | |  |
| **POC VL** | >400  Sensitivity (95% CI) | <400  Specificity (95% CI) |  |
| >400 | 9 | 2 | 11 |
| <400 | 6 | 224 | 230 |
| Total | 15  60.0% (32.3, 83.7) | 226  99.1% (96.8, 99.9) | 241  96.7% (93.6, 98.6) |
| **VL>1000** | | | |
|  | **SOC VL** | |  |
| **POC VL** | >1000  Sensitivity (95% CI) | <1000  Specificity (95% CI) |  |
| >1000 | 7 | 2 | 9 |
| <1000 | 5 | 227 | 232 |
| Total | 12  58.3% (27.7, 84.8) | 229  99.1% (96.9, 99.9) | 241  97.1% (94.1, 98.8) |
| **90 day (i.e., +/- 90 days, kids), n=506 samples** | | | |
| **VL>50** | | | |
|  | **SOC VL** | |  |
| **POC VL** | >50  Sensitivity (95% CI) | <50  Specificity (95% CI) |  |
| >50 | 72 | 29 | 101 |
| <50 | 105 | 300 | 405 |
| Total | 177  40.7% (33.4, 48.3) | 329  91.2% (87.6, 94.0) | 506  73.5% (69.4, 77.3) |
| **VL>200** | | | |
|  | **SOC VL** | |  |
| **POC VL** | >200  Sensitivity (95% CI) | <200  Specificity (95% CI) |  |
| >200 | 43 | 15 | 58 |
| <200 | 53 | 395 | 448 |
| Total | 96  44.8% (34.6, 55.3) | 410  96.3% (94.0, 98.0) | 506  86.6% (83.3, 89.4) |
| **VL>400** | | | |
|  | **SOC VL** | |  |
| **POC VL** | >400  Sensitivity (95% CI) | <400  Specificity (95% CI) |  |
| >400 | 37 | 11 | 48 |
| <400 | 27 | 431 | 458 |
| Total | 64  57.8% (44.8, 70.1) | 442  97.5% (95.6, 98.8) | 506  92.5% (89.8, 94.6) |
| **VL>1000** | | | |
|  | **SOC VL** | |  |
| **POC VL** | >1000  Sensitivity (95% CI) | <1000  Specificity (95% CI) |  |
| >1000 | 32 | 11 | 43 |
| <1000 | 12 | 451 | 463 |
| Total | 44  72.7% (57.2, 85.0) | 462  97.6% (95.8, 98.8) | 506  95.5% (93.3, 97.1) |
| **90 days (i.e., +/- 90 days, mamas), n= 394 unique samples** | | | |
| **VL>50** | | | |
|  | **SOC VL** |  |  |
| **POC VL** | >50  Sensitivity (95% CI) | <50  Specificity (95% CI) |  |
| >50 | 25 | 24 | 49 |
| <50 | 65 | 280 | 345 |
| Total | 90  27.8% (19.0, 38.2) | 304  92.1% (88.5, 95.0) | 394  77.4% (73.0, 81.4) |
| **VL>200** | | | |
|  | **SOC VL** |  |  |
| **POC VL** | >200  Sensitivity (95% CI) | <200  Specificity (95% CI) |  |
| >200 | 17 | 7 | 24 |
| <200 | 22 | 348 | 370 |
| Total, | 39  43.6% (27.8, 60.4) | 355  98.0% (96.0, 99.2) | 394  92.6% (89.6, 95.0) |
| **VL>400** | | | |
|  | **SOC VL** | |  |
| **POC VL** | >400  Sensitivity (95% CI) | <400  Specificity (95% CI) |  |
| >400 | 13 | 6 | 19 |
| <400 | 17 | 358 | 375 |
| Total | 30  43.3% (25.5, 62.6) | 364  98.4% (96.4, 99.4) | 394  94.2% (91.4, 96.3) |
| **VL>1000** | | | |
|  | **SOC VL** | |  |
| **POC VL** | >1000  Sensitivity (95% CI) | <1000  Specificity (95% CI) |  |
| >1000 | 10 | 5 | 15 |
| <1000 | 9 | 370 | 379 |
| Total | 19  52.6% (28.9, 75.6) | 375  98.7% (97.0, 99.6) | 394  96.4% (94.1, 98.0) |
