# Supplementary material for: Real-world performance of point-of-care vs. standard-of-care HIV viral load testing in western Kenya: Secondary analysis of Opt4Kids and Opt4Mamas studies
Source: PLOS Glob Public Health. 2024 Jun 24;4(6):e0003378. doi: 10.1371/journal.pgph.0003378 (PMC11195974; doi:10.1371/journal.pgph.0003378)
Supplement: S1 File — (PDF) [file pgph.0003378.s003.pdf]

Opt4Kids_date_diff_0

ptid	pocVL	socVL	closest_day_diff
2582	20	10	0
3009	20	10	0
3154	20	10	0
2689	20	10	0
3951	20	10	0
2007	20	10	0
3016	20	10	0
2668	20	10	0
707	83	10	0
234	20	10	0
3165	20	10	0
1618	20	10	0
448	182	99	0
1782	20	10	0
1886	20	10	0
3217	20	10	0
2512	20	10	0
1890	20	10	0
758	20	10	0
1470	20	10	0
3427	20	10	0
2694	20	10	0
2501	20	10	0
3046	20	10	0
1107	20	10	0
1731	20	10	0
1575	20	10	0
3227	48	10	0
3549	20	10	0
3725	20	10	0
2385	75	10	0
2178	20	10	0
2416	20	10	0
1272	20	10	0
1722	20	10	0
1639	20	10	0
884	20	10	0
1707	20	42	0
1305	20	10	0
2279	20	10	0
836	20	10	0
3650	20	10	0
3044	20	10	0
863	41	10	0
2120	20	10	0
1554	85	120	0

3844	20	10	0
3409	20	10	0
3638	20	10	0
2229	1950	521	0
1602	1480	10	0
1622	20	276	0
3879	203	10	0
1952	20	10	0
2540	20	10	0
2311	20	10	0
13	130	10	0
3801	20	10	0
1476	20	10	0
2711	20	10	0
2669	20	10	0
3787	20	10	0
2929	20	10	0
19	20	10	0
2963	222	10	0
1982	20	10	0
2479	20	1490	0
3180	20	78	0
3003	20	10	0
2542	20	10	0
3497	20	10	0
3208	20	10	0
2060	20	10	0
2577	20	10	0
3820	20	274	0
2484	10700	5572	0
3169	652000	231171	0
1213	20	123	0
1471	1330	1100	0
1108	20	10	0
3131	20	10	0
3408	20	64	0
393	20	10	0
3831	20	10	0
1113	20	10	0
979	20	10	0
2710	20	10	0
764	20	57	0
3900	20	106	0
2139	20	10	0
43	20	10	0
125	20	10	0
524	20	120	0
1413	83	52	0

2309	20	10	0
596	20	10	0
2780	20	10	0
2413	1600	912	0
1951	20	29	0
37	20	45	0
1813	20	10	0
71	20	10	0
3761	20	10	0
2713	20	10	0
3143	20	10	0
2322	20	10	0
618	20	10	0
3962	80	45	0
2559	272	170	0
2549	20	1740	0
3123	20	10	0
714	20	10	0
1252	20	179	0
3313	20	10	0
2092	20	10	0
755	20	10	0
1403	20	33	0
1216	20	88	0
2579	20	10	0
1312	20	10	0
1519	20	10	0
2095	90	90	0
3530	20	39	0
3631	20	10	0
3099	20	10	0
25	20	10	0
2615	13400	29900	0
3179	145	103	0
1606	20	10	0
2307	136	297	0
1626	20	308	0
3839	20	39	0
3013	20	258	0
1094	275	663	0
3662	20	43	0
2109	20	10	0
3058	20	10	0
3980	20	630	0
3270	20	169	0
377	20	200	0
1168	20	95	0
3085	2780	1470	0

3268	20	10	0
2379	20	57	0
3076	20	291	0
3777	20	10	0
1892	20	66	0
2533	20	93	0
3915	20	20	0
2166	2620	2850	0
2453	20	10	0
309	20	10	0
2508	20	28	0
2499	20	131	0
3040	20	628	0
122	76	66	0
2627	60	152	0
1384	20	10	0
2419	20	93	0
3279	20	203	0
756	20	101	0
1159	20	28	0
68	20	217	0
2085	20	132	0
3214	20	471	0
82	20	38	0
3356	20	144	0
749	20	31	0
3702	190	350	0
966	20	216	0
1018	20	244	0
862	20	94	0
3744	20	797	0
84	20	110	0
2009	20	10	0
2306	20	513	0
968	20	52	0
1382	20	10	0
2129	20	10	0
1975	20	148	0
923	20	10	0
2924	20	268	0
192	20	10	0
199	20	10	0
2753	20	103	0
1808	20	30	0
3627	20	10	0
1151	20	10	0
2225	20	220	0
3393	20	258	0

1703	20	10	0
1026	20	27	0
2955	20	10	0
3877	2650	1910	0
2555	20	10	0
1500	20	10	0
2671	20	10	0
1105	20	10	0
3768	375	10	0
2073	2580	4040	0
2455	20	10	0
1110	39100	6189	0
3531	20	10	0
498	5543	6009	0
1270	20	10	0
3853	20	10	0
721	20	10	0
3117	11400	2699	0
2877	129	10	0
1193	20	10	0
3896	20	10	0
2491	20	10	0
530	20	10	0

Opt4Kids_date_diff_30

ptid	pocVL	socVL	closest_day_diff
1494	20	10	9
3041	20	10	13
2582	20	10	0
3009	20	10	0
3154	20	10	0
2689	20	10	0
3951	20	10	0
875	20	10	28
2007	20	10	0
3016	20	10	0
3589	20	10	28
2668	20	10	0
1017	20	10	23
455	44	10	28
707	83	10	0
234	20	10	0
3165	20	10	0
1618	20	10	0
448	182	99	0
3741	20	108	14
3716	20	10	28
1782	20	10	0
3629	20	10	29
496	113	10	14
3222	20	10	28
1886	20	10	0
3217	20	10	0
2512	20	10	0
3863	20	10	12
578	20	10	12
1258	20	10	28
2961	20	10	28
1890	20	10	0
1024	20	10	28
758	20	10	0
1470	20	10	0
3427	20	10	0
2694	20	10	0
2501	20	10	0
3046	20	10	0
3868	20	10	28
78	20	3350	28
1107	20	10	0
1731	20	10	0
1007	107000	40840	26
1575	20	10	0

3227	48	10	0
3549	20	10	0
3725	20	10	0
1133	86	10	28
2385	75	10	0
2178	20	10	0
2489	56	104	26
2416	20	10	0
1272	20	10	0
1722	20	10	0
797	20	10	28
2006	20	10	28
1639	20	10	0
2076	20	44	25
884	20	10	0
1707	20	42	0
1433	1540	10	28
1305	20	10	0
2279	20	10	0
836	20	10	0
2608	79	10	21
1284	20	10	28
3650	20	10	0
3044	20	10	0
863	41	10	0
3977	20	10	23
886	20	536	16
1142	20	10	27
819	20	10	28
2120	20	10	0
1554	85	120	0
2324	20	10	1
3844	20	10	0
3409	20	10	0
2985	20	1548	28
3626	2470	625	15
3638	20	10	0
2229	1950	521	0
1602	1480	10	0
1622	20	276	0
3879	203	10	0
1952	20	10	0
2540	20	10	0
116	20	10	20
2311	20	10	0
13	130	10	0
3801	20	10	0
3651	20	10	2

832	49	10	7
1476	20	10	0
2711	20	10	0
2669	20	10	0
3866	20	1838831	1
3787	20	10	0
2929	20	10	0
19	20	10	0
2963	222	10	0
1982	20	10	0
1791	676	196	10
2479	20	1490	0
3180	20	78	0
3003	20	10	0
2542	20	10	0
3497	20	10	0
3208	20	10	0
2060	20	10	0
2903	20	312	3
2577	20	10	0
2709	20	31	18
3789	20	1010	2
3061	20	150	28
3820	20	274	0
2484	10700	5572	0
3169	652000	231171	0
1213	20	123	0
316	20	195	1
3184	84	71	13
1471	1330	1100	0
1108	20	10	0
3131	20	10	0
244	15600	118000	13
3408	20	64	0
1734	20	33	18
2678	20	201	19
393	20	10	0
3831	20	10	0
519	20	10	1
2571	20	10	1
1113	20	10	0
979	20	10	0
2710	20	10	0
330	20	175	25
202	20	1606	25
764	20	57	0
456	57	10	2
3900	20	106	0

2139	20	10	0
43	20	10	0
125	20	10	0
3516	20	445	24
524	20	120	0
1413	83	52	0
770	161	63	4
2309	20	10	0
596	20	10	0
2780	20	10	0
3242	11900	3036	28
2413	1600	912	0
1964	20	10	28
856	71	126	22
1951	20	29	0
37	20	45	0
1302	20	144	21
3020	1720	1080	2
1813	20	10	0
71	20	10	0
2513	20	32	12
3761	20	10	0
2713	20	10	0
3143	20	10	0
2322	20	10	0
618	20	10	0
1848	20	10	1
3962	80	45	0
577	46	40	29
611	20	110	11
2559	272	170	0
2549	20	1740	0
3123	20	10	0
714	20	10	0
3887	20	10	29
1252	20	179	0
3553	20	43	2
3313	20	10	0
1520	20	405	14
2092	20	10	0
1191	184	347	21
755	20	10	0
2038	456	817	14
574	20	10	11
3060	20	1500	14
3784	42	10	30
1403	20	33	0
3652	20	10	10

741	20	82	1
1594	7600	20400	28
2528	173	10	7
636	20	130	10
1732	20	10	24
150	20	10	6
1216	20	88	0
2579	20	10	0
1312	20	10	0
1519	20	10	0
2095	90	90	0
3530	20	39	0
2899	20	10	6
3631	20	10	0
3099	20	10	0
25	20	10	0
2328	324000	353000	14
822	102000	196000	14
2588	78	96	12
1169	20	10	14
2615	13400	29900	0
2305	20	85	30
2376	20	355	7
3179	145	103	0
1394	20	48	14
1606	20	10	0
528	4120	2570	20
2999	20	10	28
3309	20	10	25
1548	20	66	27
2151	20	134	28
2307	136	297	0
2752	51	23	8
1626	20	308	0
1585	633	2400	28
2972	20	10	28
3839	20	39	0
3013	20	258	0
1094	275	663	0
3662	20	43	0
1687	86	210	14
2109	20	10	0
3058	20	10	0
3980	20	630	0
3270	20	169	0
377	20	200	0
1168	20	95	0
2786	20	50	27

3085	2780	1470	0
112	166	547	13
3268	20	10	0
2379	20	57	0
2988	20	71	28
3697	20	10	28
3781	20	10	28
3076	20	291	0
3777	20	10	0
1892	20	66	0
2533	20	93	0
3915	20	20	0
825	291000	118000	1
624	20	133	23
2166	2620	2850	0
2453	20	10	0
3000	20	10	28
309	20	10	0
2790	1740	330	28
2508	20	28	0
2499	20	131	0
3040	20	628	0
122	76	66	0
3290	87	662	29
2627	60	152	0
1384	20	10	0
2419	20	93	0
3279	20	203	0
957	20	124	28
756	20	101	0
1159	20	28	0
68	20	217	0
2085	20	132	0
10	59	10	14
3214	20	471	0
82	20	38	0
1738	20	204	23
3647	29400	1700	15
2861	20	38	29
3356	20	144	0
749	20	31	0
3702	190	350	0
3754	43	159	7
966	20	216	0
1018	20	244	0
862	20	94	0
3744	20	797	0
1805	97	10	28

84	20	110	0
2009	20	10	0
2306	20	513	0
968	20	52	0
1382	20	10	0
2129	20	10	0
2101	61	262	23
1975	20	148	0
923	20	10	0
2924	20	268	0
192	20	10	0
199	20	10	0
1578	364	644	19
2712	20	369	28
2753	20	103	0
1808	20	30	0
3627	20	10	0
1151	20	10	0
2566	86	977	28
2225	20	220	0
3393	20	258	0
1703	20	10	0
1332	20	10	28
1026	20	27	0
2117	20	25	7
2955	20	10	0
492	158	10	28
3877	2650	1910	0
2555	20	10	0
831	20	199	4
1500	20	10	0
2671	20	10	0
1105	20	10	0
3768	375	10	0
2370	20	10	29
3331	5420	2079	1
2073	2580	4040	0
2455	20	10	0
1110	39100	6189	0
3765	61	10	1
3531	20	10	0
1469	20	10	20
3959	20	10	3
498	5543	6009	0
113	138000	10662	29
1270	20	10	0
2248	20	10	29
3064	1590	61865	15

3853	20	10	0
660	20	10	30
1478	20	10	14
721	20	10	0
3117	11400	2699	0
2877	129	10	0
1193	20	10	0
3896	20	10	0
2491	20	10	0
530	20	10	0
2177	20	10	24

Opt4Kids_date_diff_90

ptid	pocVL	socVL	closest_day_diff
1800	20	10	70
1494	20	10	9
2600	20	10	58
1136	20	10	38
1572	19100	10	56
3041	20	10	13
2582	20	10	0
3009	20	10	0
3154	20	10	0
2290	20	10	52
2689	20	10	0
3951	20	10	0
2723	20	10	65
875	20	10	28
2536	20	275	34
1435	20	10	58
2680	3030	10	63
2007	20	10	0
3016	20	10	0
3589	20	10	28
2668	20	10	0
499	20	10	61
699	3042	4561	60
1017	20	10	23
455	44	10	28
707	83	10	0
234	20	10	0
3165	20	10	0
3835	20	10	82
1618	20	10	0
448	182	99	0
3741	20	108	14
2096	20	10	48
2238	20	42	31
3716	20	10	28
2391	20	10	56
1782	20	10	0
860	20	10	56
3629	20	10	29
2476	20	10	76
496	113	10	14
3222	20	10	28
1886	20	10	0
3217	20	10	0
2512	20	10	0
3863	20	10	12

578	20	10	12
3158	20	10	84
1258	20	10	28
1082	20	10	41
2961	20	10	28
1890	20	10	0
2200	20	10	90
1024	20	10	28
758	20	10	0
1034	93	10	65
1470	20	10	0
3427	20	10	0
924	20	10	58
2694	20	10	0
2501	20	10	0
3046	20	10	0
3868	20	10	28
78	20	3350	28
2130	20	10	35
1107	20	10	0
1731	20	10	0
1007	107000	40840	26
1127	757	211	66
1575	20	10	0
3227	48	10	0
3549	20	10	0
3725	20	10	0
1133	86	10	28
2385	75	10	0
2178	20	10	0
2489	56	104	26
370	20	10	84
2416	20	10	0
453	54	3850	54
1272	20	10	0
3216	20	10	36
1722	20	10	0
797	20	10	28
2006	20	10	28
133	20	10	39
1639	20	10	0
1819	20	10	56
1670	20	10	42
2076	20	44	25
884	20	10	0
1289	20	10	42
1707	20	42	0
1433	1540	10	28

274	20	10	42
1305	20	10	0
443	20	10	86
2279	20	10	0
836	20	10	0
2608	79	10	21
3138	20	10	35
892	20	10	35
1284	20	10	28
3650	20	10	0
3044	20	10	0
1006	20	10	62
863	41	10	0
3977	20	10	23
2599	61800	11554	34
1375	20	10	64
1784	45	10	47
886	20	536	16
1142	20	10	27
1569	62	10	34
819	20	10	28
2120	20	10	0
1766	201	10	40
1554	85	120	0
2324	20	10	1
3844	20	10	0
3409	20	10	0
1786	20	69	84
2985	20	1548	28
3626	2470	625	15
3638	20	10	0
2229	1950	521	0
1602	1480	10	0
1622	20	276	0
3879	203	10	0
1952	20	10	0
2540	20	10	0
116	20	10	20
2311	20	10	0
13	130	10	0
3801	20	10	0
3651	20	10	2
832	49	10	7
1476	20	10	0
2033	20	10	67
2711	20	10	0
2669	20	10	0
3866	20	1838831	1

3787	20	10	0
2929	20	10	0
19	20	10	0
2963	222	10	0
2271	20	10	70
2692	20	10	76
1982	20	10	0
2	1230	1183	36
1111	20	10	34
238	20	10	83
1533	20	10	63
3199	20	10	84
1791	676	196	10
3318	20	10	58
2479	20	1490	0
899	20	10	49
97	20	225	54
3180	20	78	0
3003	20	10	0
2292	20	67	71
2542	20	10	0
3999	20	10	66
3497	20	10	0
1963	293	134	73
2463	20	10	33
3208	20	10	0
3907	20	68	86
2060	20	10	0
738	20	10	50
2903	20	312	3
2577	20	10	0
3899	171	98	53
2709	20	31	18
3789	20	1010	2
1493	20	49	43
3061	20	150	28
1852	20	24	62
3820	20	274	0
3312	20	10	65
2484	10700	5572	0
3169	652000	231171	0
400	20	10	32
1213	20	123	0
1730	20	10	34
316	20	195	1
3184	84	71	13
3336	20	32	86
1471	1330	1100	0

1108	20	10	0
3131	20	10	0
244	15600	118000	13
3408	20	64	0
1734	20	33	18
2678	20	201	19
393	20	10	0
3831	20	10	0
519	20	10	1
2571	20	10	1
1113	20	10	0
3132	20	10	55
979	20	10	0
2710	20	10	0
3124	20	10	88
330	20	175	25
202	20	1606	25
22	20	10	56
764	20	57	0
456	57	10	2
2232	20	10	63
3900	20	106	0
2139	20	10	0
43	20	10	0
3895	1370	2817	59
125	20	10	0
3516	20	445	24
85	20	155	62
524	20	120	0
1413	83	52	0
770	161	63	4
2309	20	10	0
1694	20	10	42
596	20	10	0
2780	20	10	0
371	20	212	62
3242	11900	3036	28
2413	1600	912	0
1964	20	10	28
3557	20	10	89
3695	64400	91	49
2435	20	10	35
2421	41800	10	56
856	71	126	22
1951	20	29	0
1180	20	10	84
37	20	45	0
1302	20	144	21

3020	1720	1080	2
1813	20	10	0
71	20	10	0
2513	20	32	12
3761	20	10	0
2713	20	10	0
2408	7760	53598	45
1120	20	885	61
3143	20	10	0
2322	20	10	0
618	20	10	0
3928	20	136	56
1848	20	10	1
3962	80	45	0
577	46	40	29
611	20	110	11
2559	272	170	0
220	20	22	86
2549	20	1740	0
3123	20	10	0
714	20	10	0
3887	20	10	29
1121	20	226	61
1252	20	179	0
2687	20	10	71
3553	20	43	2
3053	20	535	83
3313	20	10	0
593	20	10	70
1520	20	405	14
2092	20	10	0
1191	184	347	21
755	20	10	0
2038	456	817	14
574	20	10	11
3060	20	1500	14
3232	20	10	32
3784	42	10	30
1403	20	33	0
703	20	10	76
3652	20	10	10
741	20	82	1
1594	7600	20400	28
2528	173	10	7
636	20	130	10
2369	919	61	77
3574	20	10	85
1732	20	10	24

3958	20	10	90
2245	20	55	56
3661	20	10	84
1535	20	125	62
1361	20	10	56
2432	20	22	85
834	20	51	48
150	20	10	6
3803	20	10	90
1216	20	88	0
2579	20	10	0
1312	20	10	0
1519	20	10	0
2095	90	90	0
751	20	39	49
3530	20	39	0
918	20	10	89
2899	20	10	6
1192	20	145	55
3631	20	10	0
3099	20	10	0
25	20	10	0
402	20	74	75
3875	44	61	60
2328	324000	353000	14
822	102000	196000	14
2588	78	96	12
3002	20	56	44
1281	20	175	84
2203	20	10	56
1169	20	10	14
1141	20	10	63
2615	13400	29900	0
2305	20	85	30
946	136	50	39
1039	20	66	52
2376	20	355	7
3179	145	103	0
1394	20	48	14
1606	20	10	0
1953	20	10	57
528	4120	2570	20
2999	20	10	28
2551	3590	31400	66
2527	20	262	66
3309	20	10	25
184	20	10	55
1548	20	66	27

2151	20	134	28
2307	136	297	0
2752	51	23	8
1745	20	8690	83
2881	20	10	77
3092	20	10	58
3377	98	10	84
1184	20	157	59
1626	20	308	0
1585	633	2400	28
2972	20	10	28
3839	20	39	0
3013	20	258	0
1094	275	663	0
3662	20	43	0
1687	86	210	14
1553	20	10	84
1185	20	10	56
2109	20	10	0
3058	20	10	0
3980	20	630	0
3270	20	169	0
377	20	200	0
1168	20	95	0
2786	20	50	27
3085	2780	1470	0
112	166	547	13
1928	20	10	74
3268	20	10	0
2379	20	57	0
2988	20	71	28
3697	20	10	28
3781	20	10	28
3076	20	291	0
3777	20	10	0
1892	20	66	0
3634	20	10	56
2533	20	93	0
3915	20	20	0
825	291000	118000	1
2647	20	71	34
624	20	133	23
2166	2620	2850	0
2453	20	10	0
3000	20	10	28
1449	20	10	84
309	20	10	0
2790	1740	330	28

2508	20	28	0
2499	20	131	0
1867	20	10	56
3040	20	628	0
122	76	66	0
2295	144	4580	56
3290	87	662	29
3492	20	10	55
2627	60	152	0
1384	20	10	0
2419	20	93	0
3279	20	203	0
3433	20	10	31
957	20	124	28
756	20	101	0
2673	20	10	31
1159	20	28	0
68	20	217	0
2085	20	132	0
10	59	10	14
799	20	171	55
3214	20	471	0
82	20	38	0
1738	20	204	23
3647	29400	1700	15
2861	20	38	29
3356	20	144	0
749	20	31	0
3702	190	350	0
3754	43	159	7
966	20	216	0
3280	20	720	33
1018	20	244	0
2946	20	70	51
2619	20	319	31
862	20	94	0
3744	20	797	0
1805	97	10	28
245	20	10	57
84	20	110	0
2009	20	10	0
2306	20	513	0
968	20	52	0
1382	20	10	0
2129	20	10	0
2101	61	262	23
169	143	10	33
1975	20	148	0

3723	20	10	72
923	20	10	0
2924	20	268	0
192	20	10	0
3048	20	10	42
2765	1270	2850	70
199	20	10	0
3637	20	10	56
1578	364	644	19
2712	20	369	28
2753	20	103	0
1808	20	30	0
850	20	10	32
3627	20	10	0
1151	20	10	0
2566	86	977	28
2225	20	220	0
3393	20	258	0
1703	20	10	0
3856	20	10	44
2516	20	151	71
1332	20	10	28
1026	20	27	0
2117	20	25	7
2955	20	10	0
492	158	10	28
3877	2650	1910	0
2555	20	10	0
831	20	199	4
1500	20	10	0
2671	20	10	0
1105	20	10	0
3768	375	10	0
2370	20	10	29
3331	5420	2079	1
3090	312	10	52
2073	2580	4040	0
1280	20	10	35
2455	20	10	0
1110	39100	6189	0
3556	20	10	49
3765	61	10	1
3531	20	10	0
1454	20	10	33
1469	20	10	20
3959	20	10	3
3285	150000	6520	35
498	5543	6009	0

113	138000	10662	29
1346	20	10	50
2868	148000	214	52
1270	20	10	0
2248	20	10	29
1560	353	7142	44
1991	20	10	87
1661	20	10	43
3064	1590	61865	15
3853	20	10	0
2119	20	96	37
2911	20	10	59
660	20	10	30
2807	20	10	70
1478	20	10	14
1586	20	10	63
721	20	10	0
3117	11400	2699	0
2877	129	10	0
3871	20	10	37
3762	20	10	83
1193	20	10	0
826	20	10	37
3896	20	10	0
3	20	104	42
2491	20	10	0
530	20	10	0
2177	20	10	24

Opt4Mamas_date_diff_0

ptid	pocVL	socVL	closest_day_diff
3278	20	10	0
195	20	43	0
466	20	10	0
3837	20	10	0
3324	20	10	0
1089	20	10	0
3671	20	10	0
1703	20	10	0
1132	57	94	0
3549	20	80	0
1432	20	55	0
726	20	10	0
713	20	10	0
2296	20	10	0
2958	20	10	0
855	20	10	0
2693	20	10	0
812	20	10	0
1764	56	10	0
928	20	10	0
284	20	10	0
3844	20	10	0
1242	57	10	0
2206	20	10	0
2500	20	10	0
2303	20	10	0
134	149	10	0
1855	20	10	0
3294	20	10	0
563	20	10	0
3003	20	48	0
3850	20	10	0
770	20	10	0
2709	20	10	0
2082	20	66	0
1430	20	10	0
1359	20	10	0
2557	20	10	0
628	20	51	0
2589	20	10	0
1380	20	10	0
1016	132000	74200	0
2161	20	10	0
50	20	59	0
3781	20	10	0
2725	20	10	0

2041	20	10	0
3288	2550	1019	0
3616	20	10	0
2052	20	10	0
1230	81	10	0
3675	20	10	0
2203	20	10	0
1253	20	10	0
1832	20	10	0
2116	223	1180	0
2331	20	32	0
2084	48	10	0
1286	45	10	0
1704	20	10	0
1720	20	10	0
3313	20	10	0
85	20	10	0
1323	20	10	0
3961	20	10	0
2044	20	10	0
649	20	10	0
2828	20	10	0
1713	20	145	0
1633	20	10	0
2865	20	149	0
289	20	10	0
1927	20	10	0
2173	20	10	0
30	20	133	0
1452	20	10	0
1872	20	10	0
3302	89	119	0
1056	20	10	0
2406	20	10	0
150	20	171	0
2313	20	266	0
2717	20	10	0
3888	20	10	0
3791	41	10	0
1127	20	144	0
1601	197	195	0
1124	20	10	0
2971	20	10	0
1184	20	10	0
3318	20	10	0
3215	20	10	0
159	20	10	0
1746	20	10	0

898	20	10	0
2536	20	10	0
475	20	10	0
2130	20	10	0
1042	20	10	0
2312	20	10	0
2785	20	10	0
1335	20	10	0
3921	20	10	0
3134	20	10	0
704	20	10	0
176	20	10	0

Opt4Mamas_date_diff_30

ptid	pocVL	socVL	closest_day_diff
3278	20	10	0
195	20	43	0
466	20	10	0
3837	20	10	0
1254	20	10	12
1574	178	10	11
3324	20	10	0
3886	76	10	2
1089	20	10	0
2354	20	10	21
3671	20	10	0
2439	20	10	28
1703	20	10	0
3002	20	10	28
1132	57	94	0
1144	6610	10	20
2011	20	10	16
3549	20	80	0
1875	20	10	14
1432	20	55	0
726	20	10	0
1700	20	10	29
713	20	10	0
2296	20	10	0
1623	20	10	20
3860	20	10	27
1792	20	10	27
2759	20	10	22
3	98	10	8
2958	20	10	0
100	20	10	27
855	20	10	0
3683	56	10	21
2693	20	10	0
2014	20	10	27
795	20	10	1
1211	68	10	7
812	20	10	0
1833	20	10	27
3999	20	10	22
1764	56	10	0
928	20	10	0
284	20	10	0
1374	48	1115	4
1331	20	10	28
1766	20	60	28

2237	20	10	18
398	20	10	28
3844	20	10	0
1242	57	10	0
1245	20	4786	28
2206	20	10	0
2500	20	10	0
2303	20	10	0
1811	20	10	29
134	149	10	0
1855	20	10	0
3294	20	10	0
563	20	10	0
788	523	689	26
1149	20	10	29
2528	79400	96000	28
2883	20	45	28
3782	20	10	28
2926	20	10	22
3003	20	48	0
1203	20	49	13
1024	20	10	26
3850	20	10	0
2715	20	10	23
1534	20	125	9
895	20	27	30
770	20	10	0
2974	20	37	28
1588	20	71	16
200	20	10	3
2709	20	10	0
2082	20	66	0
2420	20	187	28
1430	20	10	0
203	20	10	8
2009	20	10	1
1359	20	10	0
2408	20	163	28
1446	20	10	26
2557	20	10	0
628	20	51	0
3268	20	10	28
328	44	281	28
107	20	10	20
642	20	157	14
886	20	10	2
3033	67	41	27
2589	20	10	0

1380	20	10	0
1016	132000	74200	0
869	20	10	14
2161	20	10	0
50	20	59	0
3781	20	10	0
2725	20	10	0
2041	20	10	0
3288	2550	1019	0
3616	20	10	0
879	20	10	6
2052	20	10	0
3756	20	10	24
3766	20	10	1
3573	42	79	24
1230	81	10	0
2860	20	10	29
3675	20	10	0
3903	20	10	8
2897	20	10	27
2203	20	10	0
1253	20	10	0
3295	86	4100	28
2501	20	91	29
1039	30900	30487	6
1832	20	10	0
2741	20	112	6
408	20	10	28
2187	6.7	62	29
2116	223	1180	0
2331	20	32	0
2084	48	10	0
2602	20	10	20
1145	20	147	28
2007	20	10	24
1286	45	10	0
220	20	10	30
1704	20	10	0
507	20	87	15
1720	20	10	0
657	64	55	6
3591	20	10	26
3313	20	10	0
3710	20	10	16
1816	20	10	24
85	20	10	0
1323	20	10	0
3981	20	104	8

866	20	71	28
442	20	10	22
3961	20	10	0
840	20	857	28
2044	20	10	0
1302	20	175	28
3798	20	10	6
649	20	10	0
3292	46300	253000	9
2651	20	64	1
2828	20	10	0
1713	20	145	0
1633	20	10	0
2402	20	10	30
2569	20	38	5
2357	406000	410000	29
3893	20	97	30
2865	20	149	0
1520	574	600	25
289	20	10	0
1927	20	10	0
808	20	27	28
2606	20	10	30
2150	20	10	21
2042	20	10	27
2173	20	10	0
390	79	10	14
3977	3170	2890	8
30	20	133	0
2499	20	137	8
806	20	10	30
344	20	202	19
1452	20	10	0
3624	20	10	28
1872	20	10	0
3883	20	25	14
3302	89	119	0
1458	20	10	28
2156	20	10	6
1056	20	10	0
2406	20	10	0
150	20	171	0
1617	20	10	28
3740	20	10	28
2313	20	266	0
1373	20	286	27
2717	20	10	0
3291	20	10	28

3008	20	20	28
3888	20	10	0
1193	20	10	29
3791	41	10	0
1127	20	144	0
1601	197	195	0
2090	20	10	28
1783	20	10	28
1261	20	38699	30
1902	20	10	28
804	20	10	26
443	20	10	2
1124	20	10	0
2971	20	10	0
1184	20	10	0
3318	20	10	0
3099	20	265	18
2019	20	10	28
3380	20	10	20
3231	20	10	19
1275	20	10	8
1987	20	10	5
3215	20	10	0
159	20	10	0
1489	20	10	22
1746	20	10	0
2797	20	10	26
898	20	10	0
101	65	121	5
3452	20	42	29
2536	20	10	0
475	20	10	0
2130	20	10	0
2048	20	10	29
2102	2560	184	27
3912	20	10	28
1512	20	10	29
1042	20	10	0
2459	70	10	18
1970	20	10	14
2312	20	10	0
2785	20	10	0
2992	20	10	3
2853	20	10	12
1335	20	10	0
1433	20	10	28
3921	20	10	0
3134	20	10	0

3866	20	10	27
704	20	10	0
176	20	10	0

Opt4Mamas_date_diff_90

ptid	pocVL	socVL	closest_day_diff
1831	59	44	31
3278	20	10	0
3153	20	10	70
195	20	43	0
466	20	10	0
3837	20	10	0
25	20	10	69
2137	20	10	31
1254	20	10	12
2896	257	10	53
1028	20	10	56
1574	178	10	11
171	20	10	62
3324	20	10	0
3886	76	10	2
945	114	10	62
1635	454	105	35
1089	20	10	0
2574	20	10	53
2354	20	10	21
1158	20	10	84
3671	20	10	0
3351	20	10	42
2439	20	10	28
3587	20	10	42
1703	20	10	0
2228	20	10	70
3180	20	10	37
3002	20	10	28
1132	57	94	0
1144	6610	10	20
3960	20	10	38
2011	20	10	16
2629	20	10	57
3549	20	80	0
1875	20	10	14
3993	20	10	54
1432	20	55	0
600	20	10	33
726	20	10	0
1700	20	10	29
787	20	10	53
713	20	10	0
2296	20	10	0
1623	20	10	20
2365	20	10	49

3860	20	10	27
2604	20	10	71
3834	20	10	50
396	219	10	36
1792	20	10	27
2759	20	10	22
3	98	10	8
2982	20	10	57
2958	20	10	0
1805	20	10	70
3880	378	4384	42
978	20	10	38
100	20	10	27
813	20	10	54
855	20	10	0
2819	20	10	69
2410	20	10	57
2063	20	10	39
799	76	10	64
3683	56	10	21
1379	85	10	56
3952	20	10	54
2693	20	10	0
2061	20	10	32
3420	272000	39287	61
2014	20	10	27
1952	20	10	57
438	4040	1070	85
3337	41600	89841	34
664	20	10	58
795	20	10	1
3701	20	10	64
393	20	10	62
1211	68	10	7
812	20	10	0
1833	20	10	27
3999	20	10	22
1826	20	10	77
1764	56	10	0
928	20	10	0
845	20	10	84
284	20	10	0
2718	20	10	63
1374	48	1115	4
1331	20	10	28
1766	20	60	28
2237	20	10	18
321	20	10	46

3819	20	10	88
398	20	10	28
3844	20	10	0
82	20	10	41
1242	57	10	0
1245	20	4786	28
2206	20	10	0
424	20	10	56
2500	20	10	0
2303	20	10	0
1811	20	10	29
134	149	10	0
3424	20	10	56
1855	20	10	0
2985	20	305	84
3294	20	10	0
563	20	10	0
1190	20	10	62
1337	20	10	57
788	523	689	26
1149	20	10	29
2528	79400	96000	28
2883	20	45	28
3782	20	10	28
2926	20	10	22
223	20	10	88
3003	20	48	0
2638	20	98	31
1203	20	49	13
3139	20	117	57
1024	20	10	26
3850	20	10	0
2715	20	10	23
1534	20	125	9
895	20	27	30
770	20	10	0
1179	20	10	57
890	159	10	84
2974	20	37	28
1588	20	71	16
200	20	10	3
3713	20	10	84
2709	20	10	0
3951	20	10	55
3259	20	10	52
3388	20	10	90
3527	20	10	63
2082	20	66	0

2420	20	187	28
1430	20	10	0
1982	20	24	87
203	20	10	8
2688	20	10	32
2009	20	10	1
432	20	10	52
1359	20	10	0
3628	20	10	77
2408	20	163	28
1446	20	10	26
2557	20	10	0
2999	20	108	58
1640	20	104	65
628	20	51	0
3268	20	10	28
328	44	281	28
107	20	10	20
1112	20	10	84
642	20	157	14
886	20	10	2
48	20	10	82
3033	67	41	27
2589	20	10	0
1744	60	10	76
86	2380	503	53
1380	20	10	0
1016	132000	74200	0
3998	20	67	42
3489	20	491	53
869	20	10	14
814	20	10	55
2161	20	10	0
2806	20	10	41
3542	20	78	61
864	20	10	84
1957	20	10	55
50	20	59	0
583	20	10	89
3781	20	10	0
2725	20	10	0
2041	20	10	0
3288	2550	1019	0
3616	20	10	0
879	20	10	6
2052	20	10	0
3756	20	10	24
1123	20	10	35

3766	20	10	1
3573	42	79	24
1230	81	10	0
2860	20	10	29
3675	20	10	0
3903	20	10	8
2897	20	10	27
2893	20	10	45
2203	20	10	0
1253	20	10	0
3295	86	4100	28
2685	20	10	69
2501	20	91	29
1039	30900	30487	6
208	20	10	53
1832	20	10	0
2741	20	112	6
2073	20	189	84
408	20	10	28
1303	20	38	84
2726	20	10	57
2165	20	46	70
2187	6.7	62	29
3208	20	387	72
252	20	677	42
2116	223	1180	0
678	20	155	48
2331	20	32	0
1199	20	119	62
2084	48	10	0
2602	20	10	20
1145	20	147	28
1440	20	10	35
2007	20	10	24
2666	20	10	89
1286	45	10	0
220	20	10	30
1704	20	10	0
507	20	87	15
800	71000	10	36
1720	20	10	0
657	64	55	6
1002	240	3220	34
138	20	10	33
3591	20	10	26
3313	20	10	0
811	20	10	51
1352	20	56	35

3710	20	10	16
1816	20	10	24
85	20	10	0
1417	766	201	35
1426	20	10	31
1323	20	10	0
3981	20	104	8
866	20	71	28
442	20	10	22
3961	20	10	0
746	20	10	31
2279	20	418	81
840	20	857	28
2044	20	10	0
1302	20	175	28
2703	20	10	69
3798	20	10	6
649	20	10	0
3292	46300	253000	9
2651	20	64	1
2828	20	10	0
1713	20	145	0
2006	49	10	72
1633	20	10	0
2402	20	10	30
2569	20	38	5
3566	20	10	42
2357	406000	410000	29
1138	20	706	42
3893	20	97	30
2250	20	347	85
2865	20	149	0
341	20	67	35
3780	20	10	34
1520	574	600	25
242	20	72	56
276	20	40	82
289	20	10	0
608	20	475	50
3746	20	10	56
805	20	10	38
1927	20	10	0
1177	43	1060	56
3504	20	10	56
808	20	27	28
2606	20	10	30
2150	20	10	21
2935	20	87	45

2042	20	10	27
2173	20	10	0
390	79	10	14
3977	3170	2890	8
30	20	133	0
1983	20	10	72
2499	20	137	8
806	20	10	30
344	20	202	19
1452	20	10	0
3624	20	10	28
2529	20	60	81
1872	20	10	0
3883	20	25	14
479	20	10	45
1159	20	700	70
3302	89	119	0
1458	20	10	28
1717	20	10	40
2156	20	10	6
1056	20	10	0
2406	20	10	0
150	20	171	0
1617	20	10	28
3740	20	10	28
2313	20	266	0
1373	20	286	27
2717	20	10	0
3291	20	10	28
3008	20	20	28
3888	20	10	0
1193	20	10	29
109	20	50	41
3240	20	34	33
3791	41	10	0
1127	20	144	0
361	20	462	49
1601	197	195	0
2090	20	10	28
1783	20	10	28
3783	20	10	69
3729	20	57	57
1261	20	38699	30
2653	20	10	58
2856	20	49	85
3473	20	10	77
591	20	10	77
1902	20	10	28

34	20	6608	68
576	160	10	56
3290	20	10	60
804	20	10	26
443	20	10	2
1124	20	10	0
2971	20	10	0
3309	20	10	35
1184	20	10	0
2833	20	10	73
1040	20	10	88
3318	20	10	0
3099	20	265	18
2768	20	10	84
2019	20	10	28
3380	20	10	20
3231	20	10	19
1275	20	10	8
1987	20	10	5
3260	20	10	70
40	17700	10	56
3317	20	10	70
3215	20	10	0
2000	20	10	35
3902	20	10	56
159	20	10	0
1489	20	10	22
1746	20	10	0
2797	20	10	26
898	20	10	0
101	65	121	5
3452	20	42	29
980	20	10	72
1295	20	10	77
2536	20	10	0
1519	20	10	65
475	20	10	0
2130	20	10	0
1400	20	10	85
2048	20	10	29
2102	2560	184	27
3912	20	10	28
1369	20	10	63
1512	20	10	29
1042	20	10	0
2459	70	10	18
1970	20	10	14
2312	20	10	0

2785	20	10	0
2452	20	10	63
2992	20	10	3
111	20	10	43
2853	20	10	12
1335	20	10	0
1433	20	10	28
3921	20	10	0
3134	20	10	0
3866	20	10	27
704	20	10	0
176	20	10	0
